# A Novel Infrared Thermography Sensing Approach for Rapid, Quantitative Assessment of Damage in Aircraft Composites

**DOI:** 10.3390/s20154113

**Published:** 2020-07-24

**Authors:** Spyridoula Farmaki, Dimitrios A. Exarchos, Ilias K. Tragazikis, Theodore E. Matikas, Konstantinos G. Dassios

**Affiliations:** Mechanics, Smart Sensors & Nondestructive Evaluation (MSS-NDE) Laboratory, Department of Materials Science and Engineering, University of Ioannina, GR-45110 Ioannina, Greece; s.farmaki@uoi.gr (S.F.); d.exarchos@uoi.gr (D.A.E.); i.tragazikis@uoi.gr (I.K.T.); kdassios@uoi.gr (K.G.D.)

**Keywords:** infrared thermography, damage assessment, aircraft composites, nondestructive evaluation, lock-in thermography

## Abstract

The current necessity of the scientific and industrial community, for reduction of aircraft maintenance cost and duration, prioritizes the need for development of innovative nondestructive techniques enabling fast and reliable defect detection on aircraft fuselage and wing skin parts. Herein, a new low-cost thermographic strategy, termed Pulsed Phase-Informed Lock-in Thermography, operating on the synergy of two independent, active infrared thermography techniques, is reported for the fast and quantitative assessment of superficial and subsurface damage in aircraft-grade composite materials. The two-step approach relies on the fast, initial qualitative assessment, by Pulsed Phase Thermography, of defect location and the identification of the optimal material-intrinsic frequency, over which lock-in thermography is subsequently applied for the quantification of the damage’s dilatational characteristics. A state-of-the-art ultra-compact infrared thermography module envisioned to form part of a fully-automated autonomous nondestructive testing inspection solution for aircraft was conceived, developed, and tested on aircraft-grade composite specimens with impact damages induced at variable energy levels and on a full-scale aircraft fuselage skin composite panel. The latter task was performed in semi-automated mode with the infrared thermography module mounted on the prototype autonomous vortex robot platform. The timescale requirement for a full assessment of damage(s) within the sensor’s field of view is of the order of 60 s which, in combination with the high precision of the methodology, unfolds unprecedented potential towards the reduction in duration and costs of tactical aircraft maintenance, optimization of efficiency and minimization of accidents.

## 1. Introduction

In aircraft maintenance, inspection and quantification of damage by nondestructive testing (NDT) techniques, is currently the most reliable route not only towards the identification of superficial and—undetectable by visual inspection—internal defects in the airframe structure, but also for assessing the extent of repair work required for extending the operational lifetime of the structures [1,2,3]. Federal Administration Aviation (FAA) and European Aviation Safety Agency (EASA) regulations require aerospace industries to inspect, by reliable and efficient NDT techniques, all aircraft components for possible defects and flaws, at regular intervals before and during their service life. Specifically, aircraft airworthiness requires maintenance of the aircraft, based on Original Equipment Manufacturer (OEM) specifications at three main levels, termed A-, C- and D-checks, depending on aircraft age, hours in flight, and number of take-off/landing cycles; each check involves bundles of standardized tasks. While typical A-checks, carried out every 8 to 10 weeks depending on aircraft type, involve little or no NDT inspection, C- and D-checks every 2 and 6 years, respectively, involve meticulous and painstaking NDT inspections, up to the full inspection of fuselage skin and wing skin surface for defects and damages. These inspections are currently carried out manually. They usually last 3 to 6 weeks, and can each cost from $150–$180 K (C-check) to $1.6–$1.8 M (D-check) for an aircraft like Airbus A320-200 [4]. While aircraft maintenance expenditure typically accounts for 20% of the total operating costs, ineffective inspection can hinder scheduling and reduce labor productivity by at least 50% [5,6]. Most importantly, failure to detect flaws due to inefficient inspection may lead to aircraft failure and eventually massive life loss, such as the crash of China Airlines Flight 611 due to inadequate maintenance after a tail strike incident, which costed the lives of all 225 passengers and crew on board.

Global economy forces industries to adopt accurate and—most importantly—rapid NDT inspection techniques, especially for C- and D-checks, involving minimal manpower and error. Automated techniques will increase the reliability of damage inspection and will reduce accidents and human life loss while decreasing the cost and duration of aircraft maintenance [7,8,9,10]. Infrared thermography (IRT), one of the newest nondestructive technologies, has proven exceptionally reliable, fast and cost-effective for superficial and subsurface defect detection in a wide range of mechanical systems and materials, allowing frequent and efficient maintenance with minimal manpower involvement [11,12,13,14]. IRT offers noncontact wide-area detection of subsurface defects by analyzing the information contained in energy waves radiated from the material; it can be operated in stand-alone mode or complementary to other inspection technologies [15,16]. Owing to their intrinsically high emissivity and low reflectivity, IRT inspection is exceptionally attractive for inspection of composite materials. This material class offers superior specific strengths over conventional metals and alloys and is currently seeing a tremendous increase in usage in commercial aircraft. Composite materials are trending to comprise up to more than 50% of the total aircraft structure [17,18,19]. This has given rise to extensive interest in using IRT for detecting defects such as matrix cracks, delaminations, and impact damages, in these materials [20]. The main disadvantage of thermographic methods is that they cannot efficiently tackle the influence of strong noise, and the resolution enhancement of defect detection remains critical. Το address this problem, algorithms have been used to represent the sparse and noise modeled as a mixture of Gaussian (MoG) distributions [21], on the stepped thermography approach [22] or have used the eddy current pulse-compression thermography (ECPuCT), combining the Barker code modulated eddy current excitation and pulse-compression technique [23]. IR, with laser thermal stimulation, has also been used to detect delaminations and impact damages in carbon fiber reinforced polymers (CFRPs) [24,25]. Impact damage produced at different impact energies has been successfully detected using eddy current pulsed thermography (ECPT) [26]. There are several studies dealing with detection of impact damage with lock-in thermography (LT) and also the reconstruction of the evolution of impact damage in-depth [27,28,29,30]. Benchmarking of thermographic results is commonly based on ultrasonic C-scan techniques [31].

Currently available IRT approaches are categorized into passive and active ones, depending on thermal stimulation requirements. In passive IRT, the structure or section under investigation is physically at a higher temperature than the ambient one; hence no external thermal excitation is needed for defect inspection. In active IRT, external thermal stimulation is applied in a uniform way to induce thermal contrasts to a material so that minute temperature changes can be captured [32,33,34]. The main difference between available active IRT methodologies is the modulation of the heat source; based on the type of the thermal excitation, approaches such as step heating (SH), pulsed phase thermography (PPT), vibro-thermography (VT) and LT, have been developed [35]. In the theory governing IRT, a most important type of small temperature change produced on a component’s surface is due to thermo-mechanical coupling. This coupling is mostly separated into two phenomena: reversible coupling between dilatational deformation and temperature in the solid, i.e., the *thermoelastic* effect, and dissipated thermal energy [36,37]. According to the thermodynamics of quasi-static phenomena, the change in temperature is proportional to a change in stress. Hence, the change in local temperature of the material’s surface due to heat variation is related to its internal stress field [38].

The main drawback of currently available IRT methodologies is their inability to perform fast and, at the same time, reliable quantitative inspections [39]. Among different available IRT techniques, SH, VT, and PPT can only record relative temperature variations and cannot provide information about defect depth; VT and ultrasound lock-in thermography (ULT) additionally require contact for thermal stimulation; PPT offers fast inspection and provides qualitative and quantitative phase and amplitude results at multiple frequencies, but with less signal-to-noise ratio due to a smaller energy throughput, related to the depth at which a defect can be detected [40,41,42,43]. Finally, optical LT provides quantitative amplitude and phase information at a single frequency but is time-consuming as it requires repetition at different frequencies to cover a wide range of depths [44,45,46,47]. Hence, the current state-of-the-art does not allow exploitation of infrared thermography towards satisfying the need for fast, reliable, and quantitative damage detection in aircraft composites. This work reports the development of a new thermographic approach that allows rapid and automated inspection of damage in aircraft composites, combining the advantages of different IRT techniques, allowing a reduction in inspection time by at least 30%. This would also result in a significant reduction in maintenance costs.

The novelty of the work lies in the development of an entirely new thermographic approach, based on the synergy of two complementary IRT techniques, PPT and LT, for rapid assessment of damage at all subsurface depths. The two-step approach termed Pulsed Phase-Informed Lock-in Thermography (PPI-LT), relies on the fast initial qualitative assessment of defect location by PPT, over which the optimal excitation frequency for accurate defect imaging is identified and fed as input for subsequent thorough LT inspection, for the quantitative characterization of the damage’s dilatational features. A state-of-the-art, highly powerful and versatile IRT module was developed along with custom control software, envisioned to form part of a fully automated NDT inspection solution carried by a vortex robot (VR) platform. The efficiency of the strategy and module were tested on aircraft-grade composite specimens with impact damages induced at variable energy levels and on an industrial-scale impacted composite panel simulating standard aircraft fuselage skin, complete with stiffeners, thunder-protection copper layer, and aircraft-grade paint finishing. The total timescale requirement for a full assessment by PPI-LT of damage within the instant field of view (IFOV) of the IR module, was in the order of 60 s [48]. This exceptionally low timescale, in combination with the material-extrinsic principles of the proposed approach—which enables applicability to other material classes—unravels not only unprecedented potential for substantial time and cost reductions in aircraft maintenance, especially C- and D-checks, but most importantly provides accurate, efficient and reliable damage inspection data for proper repair leading to increased aircraft performance, prevention of accidents and decrease of human life loss.

## 2. Experimental Study

### 2.1. Pulsed Phase-Informed Lock-in Thermography

**Figure 1:**
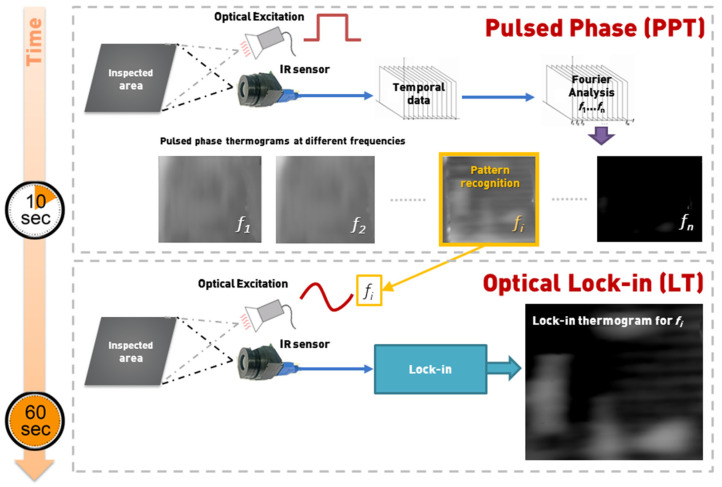
Principle of pulsed phase-informed lock-in (PPI-LT) thermographic strategy. The excitation frequency f_i_, for optimal thermographic representation of a given damage, is identified in PP-based phase 1 and fed as input for thorough dilatational analysis under lock-in configuration in phase 2.

The principle of operation of the two-phase PPI-LT strategy for fast, accurate, and qualitative damage detection in aircraft components is schematized in Figure 1. In the initial phase, the area under investigation is inspected under typical PPT wherein the heating and cooling sequence of the material under optical excitation of a square thermal pulse of variable excitation frequencies, f_1_, f_2_,…, f_n_, is captured by the IR sensor and the temporal variation of collected thermal waves is converted by fast Fourier transform (FFT), to frequency variation. This provides a series of phase and amplitude image sets (thermograms) relating to the geometric and dilatational characteristics of the defect. Pattern recognition software is employed on the set of pulsed phase phase-domain thermograms collected at different excitation frequencies f_1_, f_2_,…, f_n_, for the rapid identification of the optimal frequency, f_i_, for clearest defect depiction, based on optimum contrast features in the thermograms. This initial part, of identification of the optimal frequency f_i_ for assessing the defect, lasts approximately 10 Sec. For a series of thermographic acquisitions on the same type of material, hence the same thermal dissipation properties, the frequency sweep phase can be omitted. In the second phase of the PPI-LT strategy, the identified f_i_ is set as the frequency of sinusoidal excitation of the same area, under lock-in configuration for a longer period, to fully assess the dilatational characteristics of the damage. The lock-in thermograph collected in this stage is the sharpest and clearest possible IRT representation of the defect and contains the maximum information about damage shape and dimensions. The lock-in phase endures for approximately 50 s, depending on the value of the optimal frequency f_i_. In essence, the lock-in phase is “informed” of the optimal frequency it should operate by the pulsed phase. Overall, the synergy of PPT and LT leads to the fast and accurate characterization of damage at all subsurface depths penetrable by IRT. It must be noted that no limitation is imposed on the type of excitation source, which can include lamps, microwaves, ultrasounds, or Eddy currents, provided the source is powerful enough for the clear thermographic representation of the defects [34,49].

### 2.2. IR Sensor and Heating Source

To account for contemporary NDT inspection needs requiring compact, versatile yet powerful equipment with high resolution and accuracy, a compact and lightweight IRT system of state-of-the-art thermographic capabilities was put together. An extremely compact and powerful shutterless sensor, operating in the longwave infrared (LWIR) band with thermal sensitivity of less than 50 mK, time-to-image of less than 1 s and resolution of 640 × 480 pixels was the core of the IRT module; complete sensor specifications are provided in Table 1. A lens with a focal length of 16.7 mm, enabling a wide field of view of 37.5° and a low ratio of F/1.25 was selected to offer a system with a wide light-gathering area. The lens was equipped with a manual focus end piece, which was adjusted for focusing each time the IRT module was set at a new distance from the surface under investigation. The outer dimensions of the complete IRT module comprising the sensor, aluminum housing and lens were 30 × 30 × 45 mm^3^, equivalent to a pair of stacked matchboxes, which in combination with an ultralight weight of 70 g, is indicative of the extreme compactness and portability of the system, ideal for reliable field measurements. Sensor calibration involved the sequential thermographic acquisition of a hot and a cold surface and was performed at the beginning of each new set of measurements. The IRT module is envisioned to form part of an autonomous automated VR-based inspection solution for automatic damage identification and repair in aircraft composites in the context of an ongoing European Commission Horizon 2020 Future and Emerging Technologies (FET) project (see Acknowledgments).

**Figure 2:**
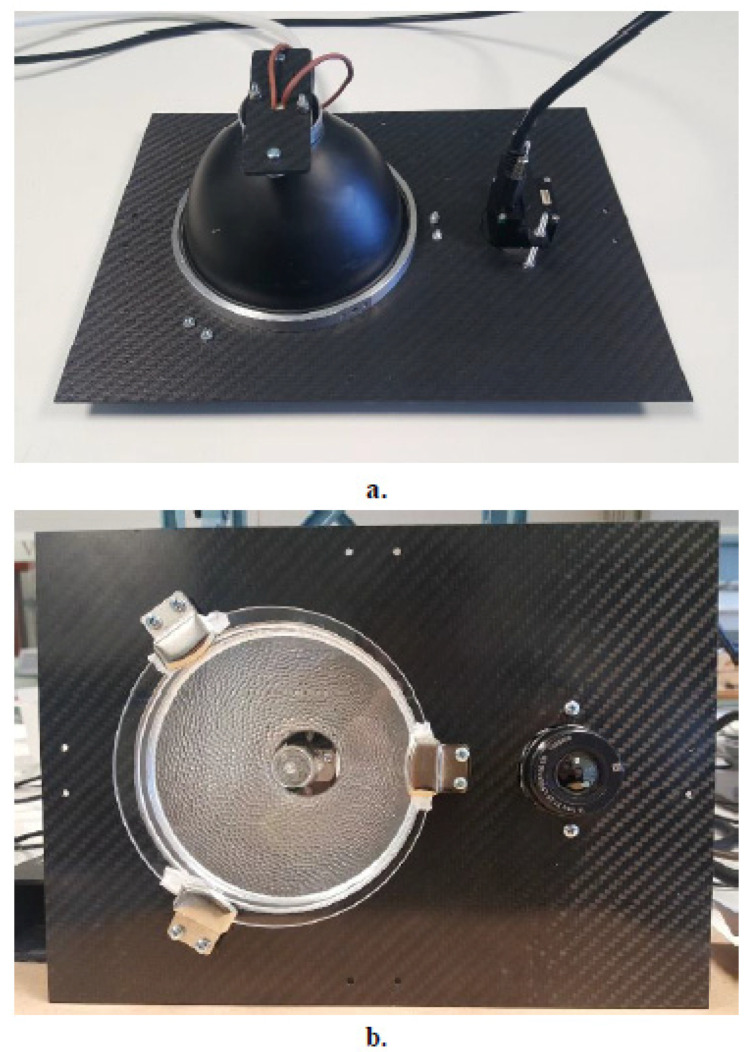
Top (**a**) and bottom (**b**) view of infrared thermography (IRT) module complete with IR sensor and excitation source with 300 W halogen lamp, IR-filtering eyepiece, and reflector resting on carbon fiber reinforced polymer (CFRP) plate. The total weight of the module including cabling is 407 g, dimensions of *ca* L × W × H: 200 × 150 × 100 mm^3^, enabling extreme compactness, portability, and maneuverability.

The selection of an appropriate thermal excitation source for continuous and uniform heat flux to the inspection area is essential for the operation of the inspection system. In this work, a powerful yet lightweight optical thermal excitation arrangement was assembled consisting of a single high-performance halogen lamp, power of 300 W, housed in an aluminum bell-shaped reflector, diameter at the opening of 80 mm, for uniform thermal energy distribution. An IR-filtering eyepiece was positioned in front of the lamp to block the reflection of IR emissions back to the sensor. The thermal excitation arrangement had a particularly important role in the overall performance of the thermographic module as it enabled the collection of high-resolution thermograms by providing uniformity and avoiding heat build-up effects on the surface of the epoxy-matrix aircraft-grade composites under investigation. The sensor and excitation sources were mounted in series on a custom-cured lightweight carbon fiber reinforced polymer (CFRP) plate, as seen in Figure 2. Accounting for extreme portability and maneuverability as required for an automated inspection solution, the total weight of the thermographic module including the mounting plate, excitation source and sensor with cabling, was 407 g with dimensions of ca. 200 × 150 × 100 mm^3^ (L × W × H). Potential degradation of the surface under investigation due to heat build-up effects on the material during excitation was excluded by measuring, with a noncontact digital infrared thermometer, the surface temperature of composite samples during a full PPI-LT acquisition cycle for a duration of 60 s with the sensor positioned at the minimum height of 250 mm from the sample surface. The maximum temperature recorded on the surface of a composite plate during a whole cycle was 34.5 °C, only a few degrees above room temperature and well below the materials’ curing temperature of 180 °C (see Section 2.4).

### 2.3. Control and Analysis Interface

A custom-developed control and analysis graphical user interface (GUI) provided for PPI-LT testing while offering a range of functions for recording, analyzing, and post-processing sequences of collected thermographic data. The lock-in frequency was determined during the initial frequency sweep phase, within the 0.001–1 Hz range relevant for aircraft-grade composite panels, by automatic identification of maximum thermal contrast in the collected phase-domain thermograms. Lock-in thermography at the selected frequency pursued. GUI capabilities include display of live thermograms, measurement of data in multi-window view, performing nonuniformity corrections of the IR sensor unit, performing calibration, setting acquisition frame rate, integration time and acquisition window limits. Thermograms can be recorded in sequence, and the results exported into various common file formats. Evaluation capabilities include performing arithmetic operations for subtracting or normalizing background, superimposing live thermograms, scaling, displaying isotherms and temperature distributions, inserting regions of interest, performing statistical analysis and pattern recognition for automatically detecting defects, and communicating with external platforms.

### 2.4. Materials and Specimens

Two types of aerospace-standard composite materials with artificially-inflicted impact damages were used to benchmark the developed PPI-LT strategy: composite specimens of variable thickness and damage induced at various impact energy levels, and an industrial-scale aircraft fuselage skin composite panel. For the latter task, the module was mounted on the prototype vortex robot platform to simulate operation in automated mode for the nondestructive inspection and repair of damage in real aircraft.

Lab-scale coupons were processed externally (see Acknowledgements), using 9 and 18 plies of unidirectional carbon reinforced epoxy resin, CYCOM 977-2-34-24KIMS-196, prepreg material of nominal cured ply thickness of 0.175 mm. Layup followed standard aircraft stacking sequence of (45/-45/90/0/90/0/90/-45/45). Autoclave curing pursued at 180 °C for 3 h. Cured laminates of thicknesses varying as per Table 2, were cut into coupons of dimensions of 100 × 150 mm^2^ with the fibers oriented along the longer dimension. Barely visible impact damages (BVID) were inflicted on the specimens by low-velocity impact at different energy levels, according to Airbus Test Method AITM1-0010 [50]. Such impacts can cause a significant amount of delamination, while the only indication of damage may be a very small, hard to spot, external surface mark. A 13 mm diameter steel hemispherical impactor was used to produce dents of depths less than 0.3 mm. The desired impact energies were achieved by varying drop height as per Table 2. At each thickness, two coupons were impacted at 8 J to observe process repeatability and confirm damage similarity.

**Figure 3:**
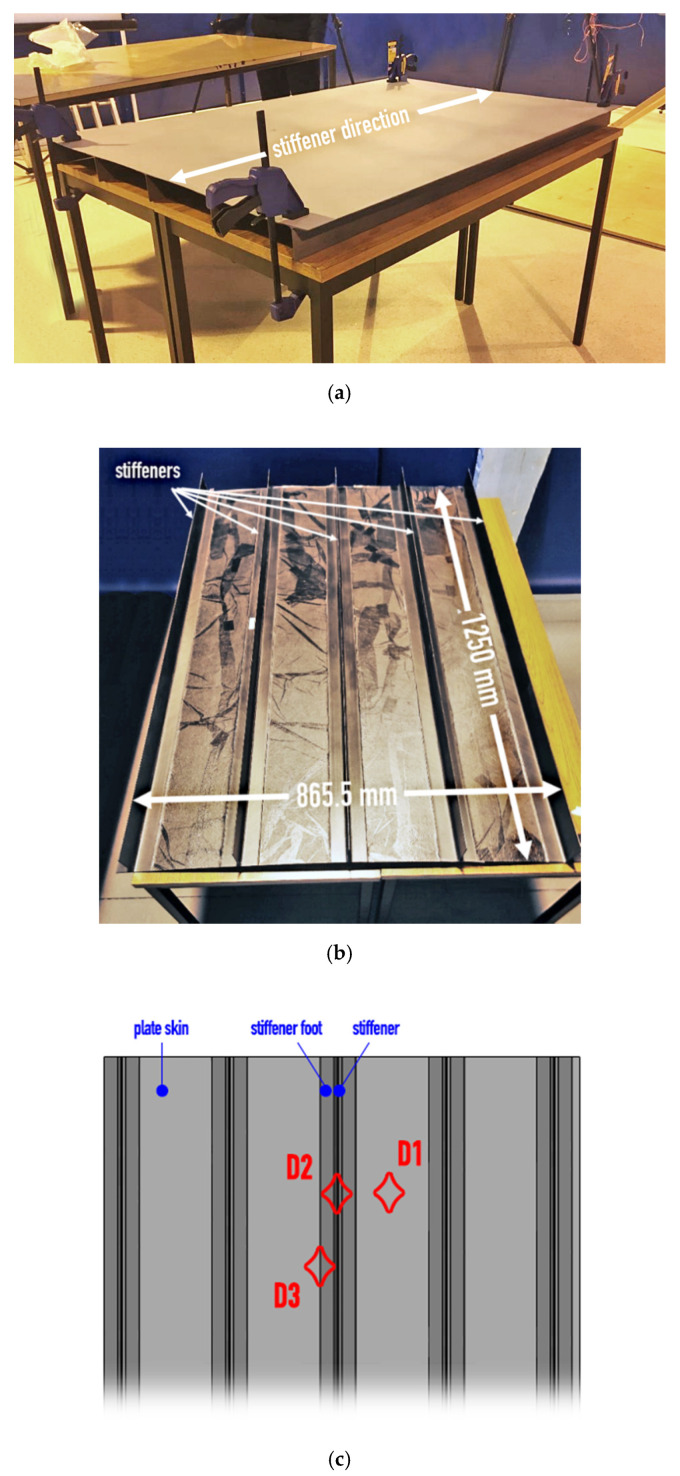
Composite flat-panel reinforced with T-stringers (**a**) front and (**b**) rear view of the panel and (**c**) schematic index of locations of barely visible impact damages.

The industrial-scale fuselage skin composite panel of dimensions of 1250 × 865.5 mm^2^ with T-shaped stiffeners was processed by an industrial expert in engineering and tooling of composite aerostructures, OPTIMAL Aerostructures (Alcabideche, Portugal) following standard fuselage skin manufacturing methodology. Stiffeners were co-cured with the skin, while a lamina of copper was applied before the carbon fiber prepreg layup to accurately reproduce the lightning strike protection solution common in composite parts for commercial and military aviation. The carbon fiber prepreg material was DeltaTech T800S-150-DT120-35, and curing followed supplier recommendations for 90 min at 120 °C, 6 bar pressure, with a linear heating ramp of 3 °C/min. Layup configurations for the skin and stiffener were [45/-45/90/0/90/0/90/-45/45] and [45/-45/90/0/90/-45/45], respectively. The panel was painted with aircraft-grade paint MIL-PRF-85285 TYPE IV-Class H, color code FS36375; the final views of the front and back sides are presented in Figure 3. Impact damages were produced at three locations on the panel using custom-built gas gun equipment. Figure 3c provides a schematic index of BVID locations. Therein, damage #1 was in a clear region of the skin far from the stiffener, damage #2 was on the stiffener-to-skin transitional area (stiffener foot), and damage #3 was exactly on the stiffener web area. Damages did not create any surface indication or dents on either side of the structure and were barely visible by an experienced naked eye.

## 3. Results and Discussion

### 3.1. Aircraft-Grade Specimens

**Figure 4:**
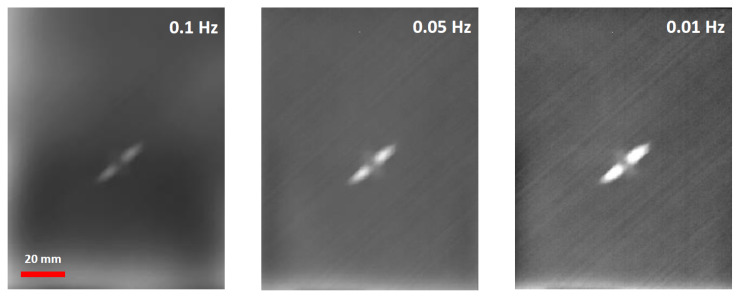
Selection of optimal frequency f_i_ in thin (9-layer) composite laminates with an impact energy of 8 Joule, under PP configuration, based on optimal contrast of phase-domain thermograms.

The initial phase of the PPI-LT thermographic strategy involved identification, under PP configuration, of the optimal frequency for the assessment of the damage by analyzing phase-domain thermographic data collected at varying frequencies. Figure 4 demonstrates three phase-domain thermograms of the same impact damage, collected with three different PP frequencies within the range of interest. In practical terms, after interrogating composite areas containing inflicted damages with the frequency sweep algorithm, automatic image analysis of phase-domain thermograms in the dataset pursued, based on the highest contrast criteria for identification of the optimal frequency f_i_ associated with the clearest depiction of damage. For the material under investigation, the value for this frequency, f_i_, was selected automatically and was found independent of the number of plies in the specimen. The invariance of f_i_ to specimen thickness indicates that the frequency is primarily material- and not geometry-intrinsic; this can be rationalized upon two factors. First, the uniform nature of specimens made for the same matrix and fibers, and, second, the uniform dilatational characteristics of impacts produced by the same impactor creating surface dents less than 0.3 mm. It is anticipated that the optimal frequency would differentiate in the case that a different material was to be interrogated.

**Figure 5:**
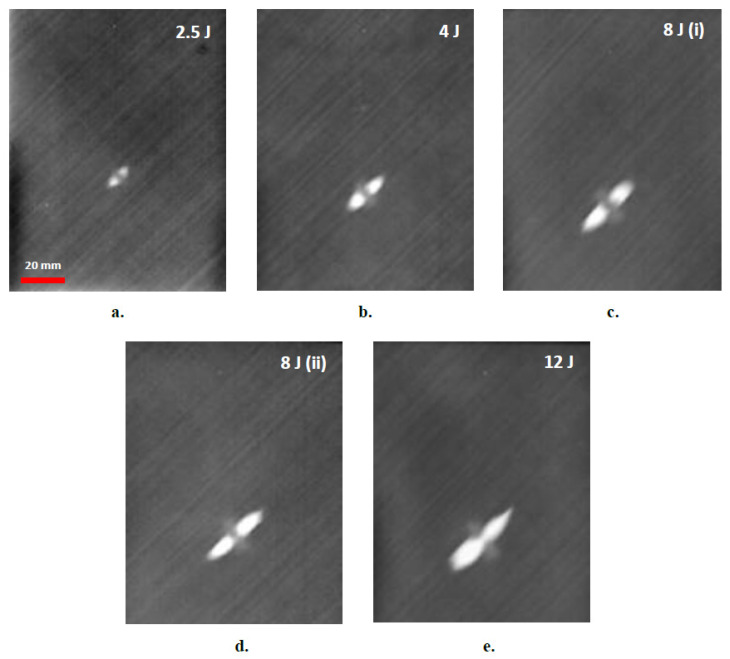
Thermographic assessment of impact damage in thin, 9-layer composite laminates for different levels of impact energies. (**a**) 2.5 J, (**b**) 4 J, (**c**) 8 J, (**d**) 8 J, (**e**) 12 J.

**Figure 6:**
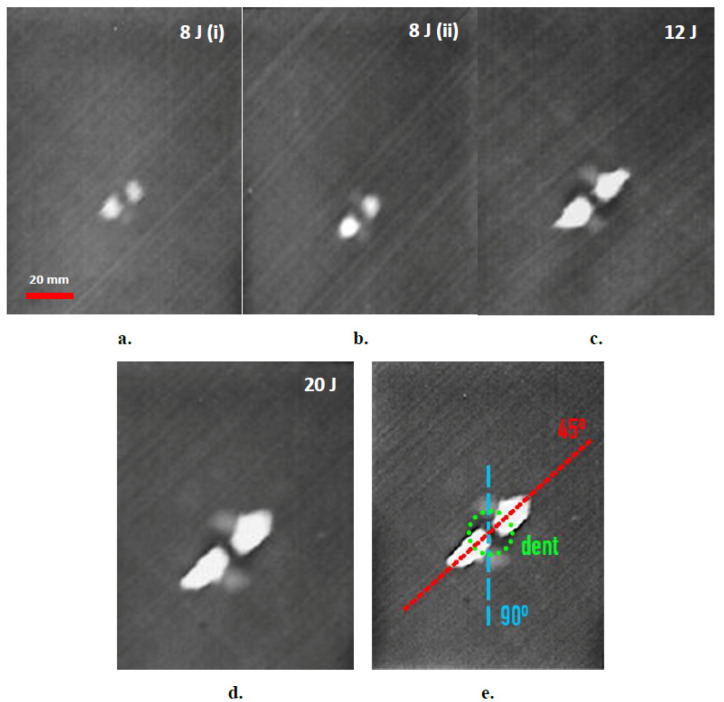
Thermographic assessment of impact damage in thick, 18-layer composite laminates for different levels of impact energies (**a**–**d**) and schematic of distinguishable damage features (**e**).

The effectiveness of the developed approach in capturing impact damages in the thin (~1.65 mm) and thick (~3.8 mm) aircraft-grade laminated specimens is demonstrated in the phase-domain thermograms of Figure 5 and Figure 6, respectively. The amount of impact energy used for each damage is included in each thermograph, while the scale bar of the first image in each subset, is common for the rest. In the thermograms, the damage is clearly depicted to vary in magnitude and size with impact energy. It is observed that the most prominent part of the thermographically distinguishable damage is the bright area along the +45° direction corresponding to fiber delamination on outer laminas, which lie on the particular orientation and were the ones most severely affected by the impact. In some cases, damage in deeper laminas is also distinguishable, albeit of smaller extent, such as damage in −45° laminas in the subset graphs of Figure 5, or damage in the 90° laminas in the thicker specimens in Figure 6. The impact dent itself, although barely visible by the naked eye, is clearly distinguishable in thermograms Figure 6c,d, as a round darker area in the middle of the damage. Figure 6e provides a schematic index of thermographically distinguishable orientations.

**Figure 7:**
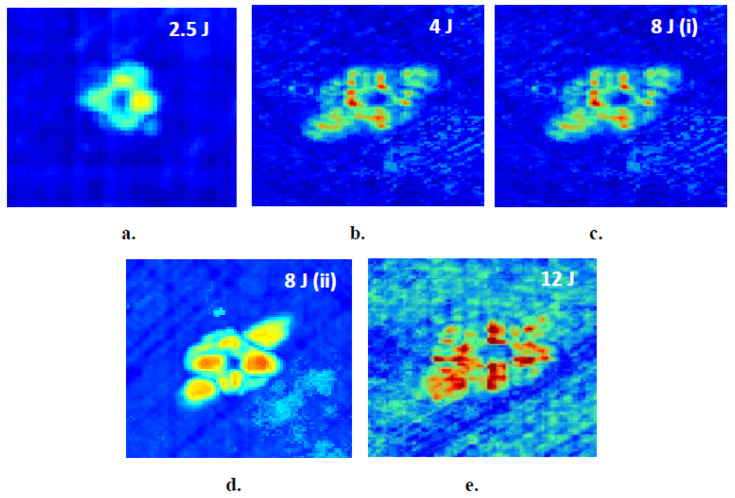
Ultrasonic C-Scan images in thin, 9-layer composite laminates for different levels of impact energies. (**a**) 2.5 J, (**b**) 4 J, (**c**) 8 J, (**d**) 8 J, (**e**) 12 J.

**Figure 8:**
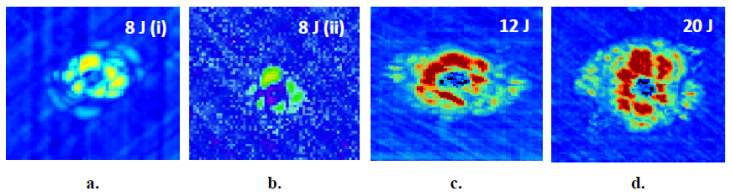
Ultrasonic C-Scan images in thick, 9-layer composite laminates for different levels of impact energies. (**a**) 8 J, (**b**) 8 J, (**c**) 12 J, (**d**) 20 J.

C-scan measurements were carried out to confirm the validity of thermographic findings. The software used for the scannings was Mistras UTWin with the following parameters: scan frequency 10 MHz, scan resolution 0.5 mm, index resolution 1 mm, and waveform sampling rate 100 MHz. Composites have an attenuation of 0.85 to 1 dB/mm, hence time gain compensation (TGC)-corrected gain was used. TGC correction could be entered either through the software or by manual input of the required gain, 1 dB/mm, as a function of depth. Initially, the front wall was set at about 80% of amplitude by changing gain and voltage, and subsequently TGC was used, so that the back-wall was at the same height. The velocity for the composite was set to 3000 m/s. The results of conventional ultrasonic C-Scan in capturing impact damages in the thin (~1.65 mm) and thick (~3.8 mm) aircraft-grade laminated specimens are shown in Figure 7 and Figure 8, respectively. On the C-scan pictures, the colors in the amplitude represent the peak amplitude of the reflected pulses with variations in color, indicating damages between layers.

**Figure 9:**
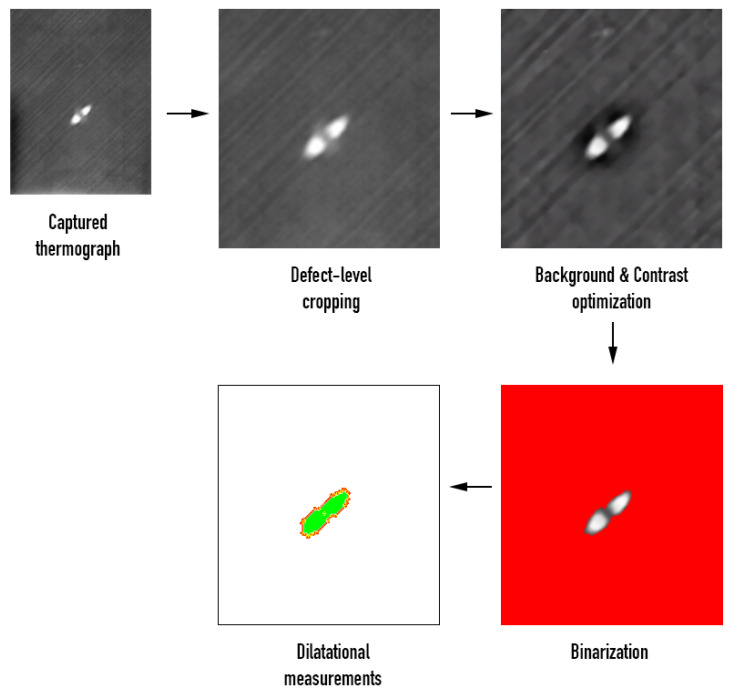
Image analysis processes for calculation of damage dimensions and area.

Image analysis on the collected phase-domain thermograms provided dilatational characteristics of the damage. Calculation of damage area and size involved a series of analytical processes, the most important of which were background correction and binarization of the image. Damage area was evaluated after transformation of the damage area, in the binary image, into a shape and calculating the number of pixels therein using the image-specific pixel-to-length conversion factor, which was introduced by aid of the scale bar. Figure 9 provides a schematic overview of the sequential processing involved in the calculation of the dilatational aspects of damage. It is therein observed that, following background correction, a dull dark halo is distinguishable around the defect. As has also been documented in previous studies [51,52,53], this zone is associated with delamination near the back face of the material, that the infrared sensor cannot fully interrogate. The more powerful—but much slower—C-scan images, Figure 7 and Figure 8, interrogate the full material thickness and validate the existence of such a round damage zone. The zone was not accounted for by the constrast-based binarization method because the information obtained by IRT from the back surface of the composite was masked by the very bright/intense damage information from the top laminas. 

**Figure 10:**
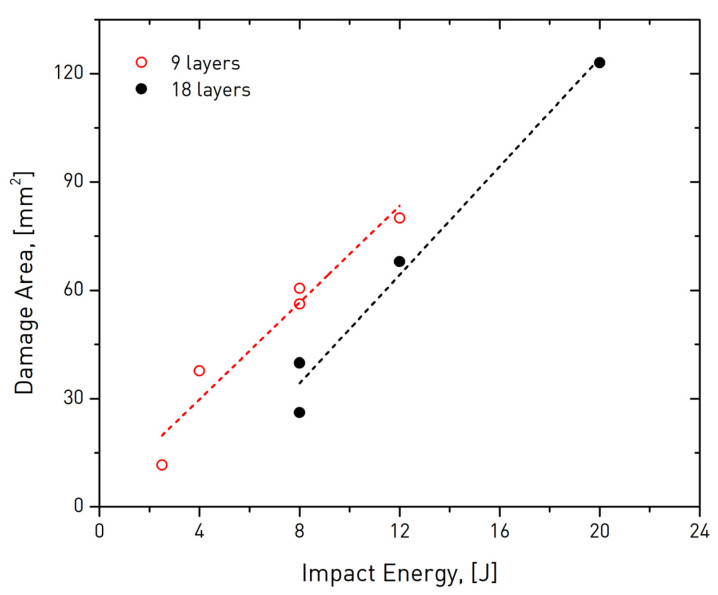
Area of damage after impact of thin (9 layer) and thick (18 layer) aircraft-grade composite specimens as a function of impact energy.

Table 3 summarizes the findings of the analysis in terms of area and perimeter of damage; the primer is plotted as a function of impact energy level in Figure 10. Therein, it is primarily observed that the extent of damage appeared to be higher in thinned specimens, a behavior that is anticipated as damage expands laterally in these specimens due to the lower material volume in the transverse direction. In 18-layer laminates, the impact wave was allowed to travel more through the (thicker) material and cause damage in larger depths, as also evidenced in the thermograms of Figure 6. The linear approximations to the data exhibited similar slopes of 6.7 and 7.5 for thin and thick specimens, respectively. This finding suggests that the rate of damage accumulation was another material- rather than geometry-intrinsic parameter.

### 3.2. Full-Scale Fuselage Skin Panel

**Figure 11:**
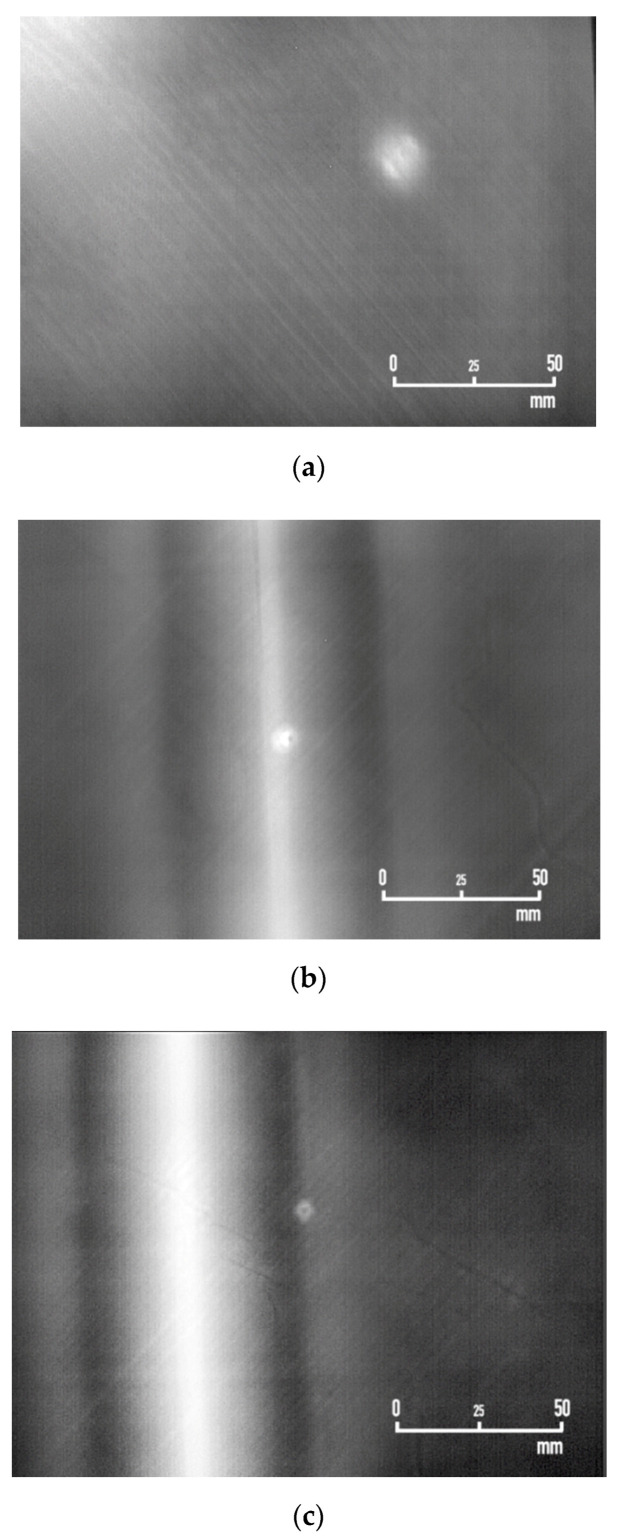
Infrared thermograms of impact damages on the industrial-scale composite panel collected by the PPI-LT strategy and module. D1 is depicted in (**a**), D2 in (**b**), and D3 in (**c**).

The industrial-scale composite fuselage skin with artificially-induced impact damages was investigated with the IRT module mounted on the prototype robotic platform envisioned to form part of an autonomous fully-automated solution for damage inspection in aircraft. The results of the assessment of the panel’s damage areas with the IRT module and PPI-LT strategy are presented in the following. Phase-domain thermograms of impact damages D1, D2, and D3 (skin region, stiffener-to-skin transitional area, and exactly of stiffener web area, respectively) on the composite panel are presented in Figure 11a–c, respectively. It is observed that PPI-LT captured damage with considerable clarity in all three cases. Image analysis for the establishment of the dilatational characteristics of damage provided respective areas of 186.17, 60.98, and 29.7 mm^2^, for D1, D2, and D3, respectively. As expected, the damage was larger in the skin region of the fuselage and minimum at the stiffener web area, where the panel was most reinforced. It is interesting to note that the field-of-view conceived in Figure 11 was established by placing the IRT module at a height of 250 mm from the fuselage skin surface. In this manner, an area of 200 × 150 mm^2^ was thermographically inspected in each acquisition. At a height of 400 mm from the surface, the field of view enlarges to 320 × 240 mm^2^. For the 60 s timescale of acquisition of one thermograph, it is estimated that the time required for complete scanning by PPI-LT of the whole fuselage of an Airbus A350-900 aircraft, with a total area of 1073 m^2^, is 9.7 days. This duration is much smaller than the one required to scan the same fuselage area by conventional ultrasonics and can be further lowered by increasing the field of view. It is entailed that the proposed PPI-LT not only lifts the tradeoff between time and reliability from which current manual inspection routes suffer but also offers unprecedented potential for fast, accurate, and efficient aircraft maintenance and repair that will prevent human live and equipment loss.

## 4. Conclusions

The development of a novel thermographic strategy for the fast, quantitative assessment of damage in aircraft composites termed Pulsed Phase-Informed Lock-in Thermography was presented. The new PPI-LT technique is based on the synergy of two complementary IRT techniques for rapid assessment of damage at all subsurface depths. The strategy involves the automatic identification and selection of an optimal excitation frequency, f_i_, for clearest defect imaging under active PP thermography configuration, which is used as input for subsequent quantitative Lock-in inspection for full dilatational characterization of damage. The frequency was found to be material-intrinsic, independent of the specimen’s thickness. A compact, lightweight, and powerful IRT module was developed along with custom control and analysis software, envisioned to form part of a future, autonomous, fully automated aircraft inspection solution carried by a vortex robot. The technique was benchmarked across aircraft-grade composite specimens impacted at variable energy levels and on an impacted aircraft fuselage skin composite panel. Dilatational analysis on phase domain thermograms revealed similar material-intrinsic behavior of the rate of damage accumulation with impact energy. Barely visible dents with underlying delamination damage as small as 11.6 mm^2^ were efficiently and straightforwardly assessed by the PPI-LT strategy. Subsurface damages in three key locations of the fuselage skin panel were also clearly captured. The potential of the technique in overcoming current aircraft C- and D-check limitations and errors leading to deadly accidents, is highlighted in view of the reliability and timescale requirements of the measurements. The proposed strategy is not limited by the nature of the material under examination and can be expanded to other types of material classes.

## Figures and Tables

**Table 1:** Specifications of the infrared thermography (IRT) sensor.

Feature	Description
Resolution	640 × 480 pixels
Material	Resistive amorphous silicon
Sensor type	Microbolometer
Cooling	Uncooled
Pixel pitch	17 µm
Spectral response (LWIR)	7–14 µm
NETD * (f/1; 300 K; 30 Hz)	<50 mK
Power consumption	<850 mW
Shutter	Shutterless
Control	Free run and External sync
Time to image	<1 s
Frame rate	Adjustable: 9–120 Hz
Connection	USB 3.0
Operating temperature range	−20–60 °C
Total weight of IR assembly (with lens)	70 g

* NETD: Noise Equivalent Temperature Difference.

**Table 2:** Aircraft-grade specimen characteristics.

Impact Energy (J)	Drop Height (mm)	Number of Coupons	Number of Layers	Cured Thickness (mm)
2.5	200	1	9	1.65
4	335	1	9	1.65
8	670	2	9	1.65
8	670	2	18	3.80
12	1000	1	9	1.65
12	1000	1	18	3.80
20	918	1	18	3.80

**Table 3:** Dilatational characteristics of damage caused on thin and thick composite specimens at different impact energy levels.

	9-Layer Laminates					18-Layer Laminates			
Impact Energy, (J)	2.5	4	8	8	12	8	8	12	20
Damage Area, (mm^2^)	11.60	37.72	60.55	56.21	80.08	26.15	39.89	67.97	123.09
Damage Perimeter, (mm)	14.42	29.05	38.88	40.28	48.31	28.54	37.08	56.58	72.53

## References

[1] D’Orazio T., Guaragnella C., Leo M., Spagnolo P. (2005). Defect detection in aircraft composites by using a neural approach in the analysis of thermographic images. NDT E Int..

[2] Avdelidis N., Hawtin B., Almond D. (2003). Transient thermography in the assessment of defects of aircraft composites. NDT E Int..

[3] Kordatos E., Dassios K., Aggelis D., Matikas T.E. (2013). Rapid evaluation of the fatigue limit in composites using infrared lock-in thermography and acoustic emission. Mech. Res. Commun..

[4] Ackert S.P. Basics of Aircraft Maintenance Programs for Financiers: Evaluation & Insights of Commercial Aircraft Maintenance Programs. http://www.aircraftmonitor.com/uploads/1/5/9/9/15993320/basics_of_aircraft_maintenance_programs_for_financiers___v1.pdf.

[5] Eurocontrol Standard Inputs for Eurocontrol Cost Benefit Analyses 2018. https://www.eurocontrol.int/sites/default/files/publication/files/standard-input-for-eurocontrol-cost-benefit-analyses-2018-edition-8-version-2.6.pdf.

[6] Palmer D. (1999). Maintenance Planning and Scheduling Handbook.

[7] Rantala J., Wu D., Busse G. (1996). Amplitude-Modulated lock-in vibrothermography for NDE of polymers and composites. Res. Nondestruct. Eval..

[8] Kelley L.P. Understanding Maintenance Caused Accidents. Proceedings of the International Society of Air Safety Investigators.

[9] Hobbs A. (2008). An Overview of Human Factors in Aviation Maintenance.

[10] Dillenz A., Busse G., Wu D. (1999). Ultrasound lock-in thermography: Feasibilities and limitations. Ind. Lasers Insp. EUROPTO Ser..

[11] Kordatos E., Aggelis D., Matikas T.E. (2012). Monitoring mechanical damage in structural materials using complimentary NDE techniques based on thermography and acoustic emission. Compos. Part B Eng..

[12] Deane S., Avdelidis N.P., Ibarra-Castanedo C., Zhang H., Yazdani-Nezhad H., Williamson A.A., Mackley T., Davis M.J., Maldague X., Tsourdos A. (2019). Application of NDT thermographic imaging of aerospace structures. Infrared Phys. Technol..

[13] Zhang H., Avdelidis N.P., Osman A., Castanedo C.I., Sfarra S., Fernandes H.C., Matikas T.E., Maldague X.P. (2017). Enhanced Infrared Image Processing for Impacted Carbon/Glass Fiber-Reinforced Composite Evaluation. Sensors.

[14] Wróbel G., Rdzawski Z. (2009). Determination of thermal diffusivity of carbon/epoxy composites with different fiber content using transient thermography. J. Achiev. Mater. Manuf. Eng..

[15] Maldague X.P.V. (2002). Introduction to NDT by active infrared thermography. Mater. Eval..

[16] Kordatos E., Exarchos D., Stavrakos C., Moropoulou A., Matikas T.E. (2013). Infrared thermographic inspection of murals and characterization of degradation in historic monuments. Constr. Build. Mater..

[17] Ciampa F., Mahmoodi P., Pinto F., Meo M. (2018). Recent Advances in Active Infrared Thermography for Non-Destructive Testing of Aerospace Components. Sensors.

[18] Hellard G. Composites in Airbus—A Long Story of Innovations and Experiences. Proceedings of the Global Investment Forum.

[19] Mrazova M. (2013). Advanced composite materials of the future in aerospace industry. INCAS Bull..

[20] Shi Z., Xu X., Ma J., Zhen D., Zhang H. (2018). Quantitative Detection of Cracks in Steel Using Eddy Current Pulsed Thermography. Sensors.

[21] Ahmed J., Gao B., Tian G., Yang Y., Fan Y.C. (2018). Sparse ensemble matrix factorization for debond detection in CFRP composites using optical thermography. Infrared Phys. Technol..

[22] Palumbo D., Cavallo P., Galietti U. (2019). An investigation of the stepped thermography technique for defects evaluation in GFRP materials. NDT E Int..

[23] Yi Q., Tian G., Malekmohammadi H., Zhu J., Laureti S., Ricci M. (2019). New features for delamination depth evaluation in carbon fiber reinforced plastic materials using eddy current pulse-compression thermography. NDT E Int..

[24] Wang Q., Hu Q., Qiu J., Pei C., Li X., Zhou H. (2020). Using differential spread laser infrared thermography to detect delamination and impact damage in CFRP. Infrared Phys. Technol..

[25] Swiderski W. (2019). Non-Destructive testing of CFRP by laser excited thermography. Compos. Struct..

[26] Liang T., Ren W., Tian G., Elradi M., Gao Y. (2016). Low energy impact damage detection in CFRP using eddy current pulsed thermography. Compos. Struct..

[27] Kersemans M., Verboven E., Segers J., Hedayatrasa S., Van Paepegem W. (2018). Non-Destructive Testing of Composites by Ultrasound, Local Defect Resonance and Thermography. MDPI Proc..

[28] Wu J., Xu C., Qi B., Hernandez F.R. (2018). Detection of Impact Damage on PVA-ECC Beam Using Infrared Thermography. Appl. Sci..

[29] Poelman G., Hedayatrasa S., Segers J., Tellez J.A.C., Van Paepegem W., Kersemans M. (2018). Optical Infrared Thermography of CFRP with Artificial Defects: Performance of Various Post-Processing Techniques. MDPI Proc..

[30] Dorafshan S., Maguire M., Collins W. (2018). Infrared Thermography for Weld Inspection: Feasibility and Application. Infrastructures.

[31] Duan Y., Zhang H., Maldague X.P., Ibarra-Castanedo C., Servais P., Genest M., Sfarra S., Meng J. (2019). Reliability assessment of pulsed thermography and ultrasonic testing for impact damage of CFRP panels. NDT E Int..

[32] Maldague X. (2001). Theory and Practice of Infrared Technology for Nondestructive Testing.

[33] Ibarra-Castanedo C., Piau J.-M., Guilbert S., Avdelidis N.P., Genest M., Bendada A., Maldague X.P.V. (2009). Comparative Study of Active Thermography Techniques for the Nondestructive Evaluation of Honeycomb Structures. Res. Nondestruct. Eval..

[34] Zalameda J., Winfree W. (2018). Detection and Characterization of Damage in Quasi-Static Loaded Composite Structures Using Passive Thermography. Sensors.

[35] Kordatos E., Exarchos D.A., Dassios K., Matikas T.E. (2014). Thermo-Electrical lockin thermography for characterization of subsurface defects. SPIE Smart Struct. Mater. Nondestruct. Eval. Health Monit..

[36] Myriounis D.P., Kordatos E., Hasan S., Matikas T.E. (2010). Crack-Tip Stress Field and Fatigue Crack Growth Monitoring Using Infrared Lock-In Thermography in A359/SiCp Composites. Strain.

[37] Mountain D.S., Webber J.M. Stress Pattern Analysis by Thermal Emission. Proceedings of the Fourth European Electro-Optics Conference.

[38] Chrysochoos A., Dupre J. (1992). An infrared set-up for continuum thermomechanics. Quant. Infrared Thermogr..

[39] Deane S., Avdelidis N.P., Ibarra-Castanedo C., Zhang H., Nezhad H.Y., Williamson A.A., Mackley T., Maldague X., Tsourdos A., Nooralishahi P. (2020). Comparison of Cooled and Uncooled IR Sensors by Means of Signal-To-Noise Ratio for NDT Diagnostics of Aerospace Grade Composites. Sensors.

[40] Maldague X., Marinetti S. (1996). Pulse phase infrared thermography. J. Appl. Phys..

[41] Maldague X., Galmiche F., Ziadi A. (2002). Advances in pulsed phase thermography. Infrared Phys. Technol..

[42] Guo X., Vavilov V.P. (2013). Crack detection in aluminum parts by using ultrasound-excited infrared thermography. Infrared Phys. Technol..

[43] Dillenz A., Zweschper T., Busse G. (2001). Progress in ultrasound phase thermography. Aerosp. Def. Sens. Simul. Control..

[44] Riegert G., Zweschper T., Busse G. (2004). Lockin thermography with eddy current excitation. Quant. Infrared Thermogr. J..

[45] Riegert G., Gleiter A., Busse G. (2006). Potential and limitations of eddy current lockin-thermography. Def. Secur. Symp..

[46] Park H., Choi M., Park J., Kim W. (2014). A study on detection of micro-cracks in the dissimilar metal weld through ultrasound infrared thermography. Infrared Phys. Technol..

[47] Tang Q., Junyan L., Yang W., Hui L. (2011). Subsurface Interfacial Defects of Metal Materials Testing Using Ultrasound Infrared Lock-in Thermography. Procedia Eng..

[48] Exarchos D.A., Dassios K., Matikas T.E. (2018). Novel infrared thermography approach for rapid assessment of damage in aerospace structures. Smart Mater. Nondestruct. Eval. Energy Syst. IV.

[49] Weekes B., Almond D.P., Cawley P., Barden T. (2012). Eddy-Current induced thermography—Probability of detection study of small fatigue cracks in steel, titanium and nickel-based superalloy. NDT E Int..

[50] Airbus S.A.S. (2005). Airbus Test Method: Determination of Compression Strength after Impact.

[51] Meola C., Boccardi S., Carlomagno G., Boffa N., Monaco E., Ricci F. (2015). Nondestructive evaluation of carbon fibre reinforced composites with infrared thermography and ultrasonics. Compos. Struct..

[52] Meola C., Boccardi S., Carlomagno G.M. (2018). A quantitative approach to retrieve delamination extension from thermal images recorded during impact tests. NDT E Int..

[53] Meola C., Boccardi S., Carlomagno G.M. (2016). Infrared Thermography in the Evaluation of Aerospace Composite Materials: Infrared Thermography to Composites.

